# Evaluation of the Tellurium Dioxide Crystal Shear Acoustic Wave Attenuation at 40–140 MHz Frequency

**DOI:** 10.3390/ma17164082

**Published:** 2024-08-16

**Authors:** Zhiyuan Mi, Huijie Zhao, Qi Guo, Yue Yu, Yaoxing Liang

**Affiliations:** 1 School of Instrumentation Science & Opto-Electronics Engineering, Beihang University, No. 37 Xueyuan Road, Haidian District, Beijing 100191, China; qguo@buaa.edu.cn (Q.G.); zb2117115@buaa.edu.cn (Y.Y.); 2 Key Laboratory of “Precision Opto-Mechatronics Technology”, Ministry of Education, No. 37 Xueyuan Road, Haidian District, Beijing 100191, China; 3 Institute of Artificial Intelligence, Beihang University, No. 37 Xueyuan Road, Haidian District, Beijing 100191, China; by2342119@buaa.edu.cn

**Keywords:** acousto-optic interaction, tellurium dioxide, attenuation, acoustic field

## Abstract

The attenuation of slow shear acoustic waves in the (110) plane of tellurium dioxide (TeO_2_) crystals was investigated. The strong acoustic anisotropy of TeO_2_ crystals results in a non-uniform acoustic power distribution, which can introduce errors in conventional acousto-optic testing methods. In this study, we propose a general method to measure the acoustic power distribution along the propagation direction of acoustic waves in non-collinear acousto-optic tunable filters (AOTFs). Additionally, we analyze the errors introduced by the non-uniform acoustic field resulting from strong acoustic anisotropy in acousto-optic testing methods. The measurements were carried out for a crystal cutoff angle of 6.5° from the [110] axis, for the ultrasound frequency range from 40 to 140 MHz. The attenuation coefficients were determined and their quadratic dependence on ultrasound frequency was confirmed.

## 1. Introduction

Acousto-optic (AO) devices, based on light diffraction by a dynamic phase grating induced in a medium by ultrasound, are widely used for spectral filtering of incoherent light [1,2,3], ultrashort laser pulse shaping [4,5], and laser beam control [4,6].

One notable AO device is the acousto-optic tunable filter (AOTF); it has many advantages, such as being electronically controlled with high speed, having a wide spectral range, and being all-solid-state. Based on the geometric relationship between the propagation directions of acoustic and optical waves within the crystal, AOTFs can be constructed in two distinct configurations: quasi-collinear and non-collinear.

In quasi-collinear AOTFs, the acoustic wave propagation direction aligns with the optical wave propagation direction. These AOTFs typically have an extremely high AO interaction length (up to 90 mm to date) to achieve an anomalously high spectral resolution. This configuration is commonly used for solving problems related to laser pulse shaping [7,8].

In non-collinear AOTFs, the acoustic wave propagation direction is approximately perpendicular to the optical wave propagation direction. The advantage of this configuration is its wide angular aperture and the effective separation of the diffracted light from the zero-order light. An important application of non-collinear AOTFs is in imaging spectral systems [2,9]. These AO devices typically have comparatively large AO crystals and apertures (up to 25 × 25 mm^2^ to date) to achieve higher light flux.

Tellurium dioxide (TeO_2_) crystal is one of the most important acousto-optic materials to date. Due to its combination of a high acousto-optic (AO) quality factor and a wide optical transparency band [10], this material is widely used in acousto-optic applications. TeO_2_ is well known for its extremely high acoustic and acousto-optic anisotropy [10,11,12].

The most commonly used mode is the slow shear wave along the [110] axis, with a slow velocity along this axis. This results in a very high AO quality factor, which is critical for acousto-optic devices, and significantly reduces the acoustic power required to achieve high diffraction efficiency. Therefore, the cut angles used for TeO_2_ in AO devices typically do not exceed 20° from the [110] axis [13].

However, in acousto-optic applications, TeO_2_ crystals are adversely affected by the high acoustic wave attenuation and the accompanying thermal effects. On the one hand, the attenuation limits the frequency range of acoustic waves that can propagate in this material (the attenuation coefficient is typically proportional to the square of the ultrasound frequency). The acoustic field intensity decreases with propagation, which also restricts the usable region of the acoustic field that can achieve high diffraction efficiency. On the other hand, acoustic wave attenuation causes heat to accumulate in the TeO_2_ crystal, resulting in temperature gradients within the crystal, which leads to distortion of its spectral response, among other issues [14,15,16].

It is known that the attenuation coefficient of the most important slow shear acoustic waves in TeO_2_ is highly direction-dependent and significantly large [10,17,18,19,20,21,22]. According to the literature [10,18,19,20], the highest attenuation is observed when the shear mode propagates along the [110] direction. At cut angles below 10°, the acoustic wave attenuation values are smaller [21,22]. Therefore, compared to other acousto-optic materials, TeO_2_ devices are typically designed to operate in a relatively low-frequency acoustic wave range to mitigate the impact of ultrasonic attenuation.

The attenuation of acoustic waves in TeO_2_ crystals is a critical parameter for practical applications. However, there is a fairly small number of papers devoted to the examination of this problem [10,17,18,19,20,21,22]. These studies have measured the attenuation values of acoustic waves along the principal axes and small cutoff angle directions at small acoustic propagation distances, but detailed measurements for the low-frequency (<150 MHz) range are even rarer. Additionally, the data reported in different studies are not entirely consistent, indicating a need for further research on low-frequency acoustic wave attenuation.

Moreover, due to the significant variation in acoustic velocity within the (110) plane of TeO_2_ crystals, the shear wave velocity ranges from a minimum of 612 m/s to a maximum of 2100 m/s, resulting in pronounced acoustic anisotropy. This high acoustic anisotropy causes the acoustic field in AO crystals to be non-uniform [23]; this phenomenon becomes more noticeable at lower frequencies and longer acoustic propagation distances. Current research has found [24,25,26,27] that the influence of acoustic beam phase structure distortion on the transmission function shape is small; the primary impact of the TeO_2_ acoustic anisotropy is due to the power redistribution in AO cells, leading to a reduction in the overall diffraction efficiency.

Typically, the testing of acoustic wave attenuation is performed using a single laser beam [18,19,20,21]. The single laser beam passes through the center height of the optical path and measures the diffraction efficiency at different propagation distances within the acoustic field. The acoustic wave power–distance relationship is calculated, and the attenuation coefficient at different frequencies is derived. The advantage of this method lies in its simplicity, convenience, and non-contact nature. However, this method is based on the plane wave acoustic field assumption, where the field intensity remains uniform and unchanged with the propagation distance in the absence of attenuation. In practice, this assumption may only be approximately valid at higher ultrasound frequencies and shorter acoustic propagation distances.

Studies have observed the complex variations in acoustic field intensity when a single laser beam is used to test AO devices [22,28], resulting from the combined effects of acoustic wave attenuation and acoustic field redistribution. Undoubtedly, acoustic field redistribution affects the measurement results of acoustic wave attenuation, potentially leading to inaccurate data. However, this influence has been overlooked in most previous studies.

This paper proposes a method for testing the amplitude distribution of the acoustic field using a full-aperture acousto-optic method. This method employs an expanded laser beam as the light source, covering the full aperture of the AO device. After passing through the acousto-optic interaction region, the full-aperture beam, carrying intensity information from different regions of the acoustic field, is projected onto a screen and captured by a camera. For non-collinear acousto-optic interaction devices, the power distribution of the slow shear wave propagating within the crystal along the propagation direction can be observed.

The proposed method can be used to measure ultrasonic attenuation in tellurium dioxide crystal, thereby avoiding the complex effects caused by a non-uniform distribution of acoustic field power. The experimental ultrasonic frequency range is 40–140 MHz, with a 6.5° cut angle in tellurium dioxide devices.

## 2. Methods

**Figure 1:**
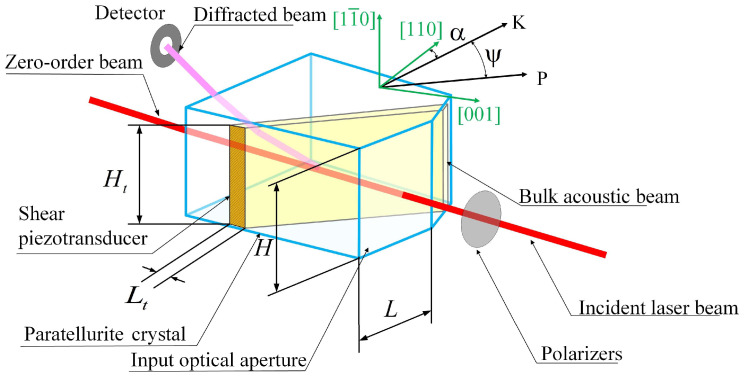
Typical non-collinear AOTF configuration.

The configuration of a non-collinear AOTF is illustrated in Figure 1. The bulk acoustic wave (BAW) vector **K** orients at an angle of *α* off the [110] crystal axis, and **P** denotes the energy flow vector, with the walk-off angle *ψ* indicating the deviation angle between the wave vector and the energy flow vector. The crystal cut angle for the examined AOTF is 6.5°, and the corresponding walk-off angle is 46.5°.

The ultrasonic beam of the slow shear BAW mode is depicted in yellow-green color. A thickness-driven transducer, welded to the side of the AO unit, is made of 41°X LiNbO_3_ plates and generates shear acoustic waves. The polarization of the shear wave is orthogonal to the acousto-optic interaction plane, which allows for better separation of the diffracted and zero-order light components.

Incident light undergoes acousto-optic interaction as it passes through the BAW region. Non-collinear acousto-optic interaction causes a polarization change in the diffracted light, while tellurium dioxide crystals have differential refractive indices for different polarization states. This results in incident and diffracted light separation. The height of the transducer determines the BAW height, while the length of the transducer determines the length of the acousto-optic interaction, which, in turn, determines the spectral resolution.

The geometric dimensions of the examined AOTF are as follows: the width and height of the input optical aperture are L=22mm and H=22mm, respectively, and the dimensions of the transducer are height $H_{t}=20\mathrm{mm}$ and length $L_{t}=3\mathrm{mm}$.

### 2.1. Acousto-Optic Interaction Basic Relations

The relationship between the transducer driving frequency and the filtered optical wavelength can be calculated with the following well-known equation [13,21]:

(1) $$f=\frac{V}{\lambda }\left(\sqrt{\left|n_{d}^{2}-n_{i}^{2}\cos\theta^{*}\right|}\pm n_{i}\sin\theta^{*}\right)$$

where f is the ultrasound frequency; V is the acoustic wave velocity (depending on *α*); λ is the optical wavelength; $\theta^{*}$ is the Bragg angle; $n_{i}$ and $n_{d}$ are the refraction indices for incident and diffracted light, respectively; and the sign depends on the polarization of incident light.

**Figure 2:**
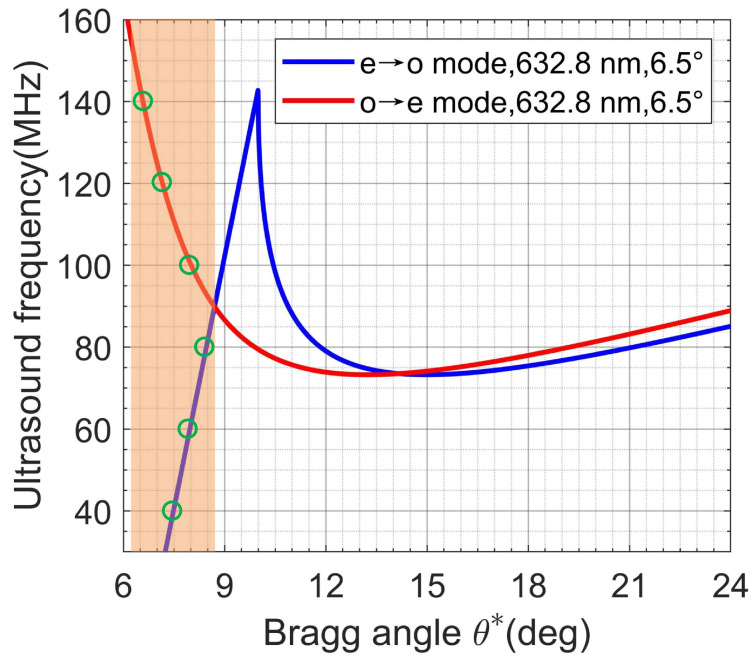
Frequency dependence of Bragg angles in paratellurite crystal for 6.5° crystal cut, green circles represents the polarization state used to measure at different ultrasound frequencies).

Based on Equation (1), a calculation was performed for the dependence of the ultrasound frequency on the Bragg angle corresponding to a 6.5° crystal cutoff angle and a wavelength of 632.8 nm from a He-Ne laser, as illustrated in Figure 2. The transducer operates within a frequency range of 40–140 MHz; thus, the calculations were primarily focused on this frequency range. For a given device, the Bragg angle for normal incident light is fixed. However, varying the angle of incidence on the AOTF allows for the Bragg angle to be adjusted.

The calculations were conducted for both the e-ray and o-ray incidences. It was determined that within the Bragg angle range of 6–9°, testing of acoustic waves across the 40–140 MHz range is feasible. Specifically, the e-ray incidence was employed for frequencies ranging from 40 to 90 MHz, while the o-ray incidence was utilized for frequencies spanning from 100 to 140 MHz. This testing approach necessitates only a minor adjustment of the Bragg angle (approximately 3°) of the device and rotation of the polarizer, which is convenient for measurements.

It is known that in the Bragg regime of AO interaction, when the phase matching condition is fulfilled, the AO diffraction efficiency is defined by

(2) $$\zeta =\frac{I_{d}}{I_{i}}=\cos^{2}\left(\frac{ql}{2}\right)$$

where ζ is the efficiency; $I_{i}$ and $I_{d}$ are the incident and diffracted light intensities, respectively; and, ql is the Raman–Nath parameter, defined as

(3) $$ql\approx \frac{2\pi \Delta n}{\lambda \cos\theta }=\frac{\pi }{\lambda \cos\theta }\sqrt{\frac{MP_{a}}{lb}}$$

Here, M is the AO quality factor, $P_{a}$ is the ultrasound power, l is the AO interaction length, and b is the acoustic beam height.

By combining Equations (2) and (3), the acoustic power passing through the acoustic field can be inferred by testing the diffraction efficiency of diffracted light. By varying the position of incidence through the acoustic field, acoustic power at different distances from the transducer can be tested. This approach enables the acquisition of the acoustic power distribution at varying distances from the transducer, which can then be used to calculate the attenuation values of acoustic waves. This constitutes the fundamental principle underlying the acousto-optic method for the assessment of acoustic attenuation coefficients.

**Figure 3:**
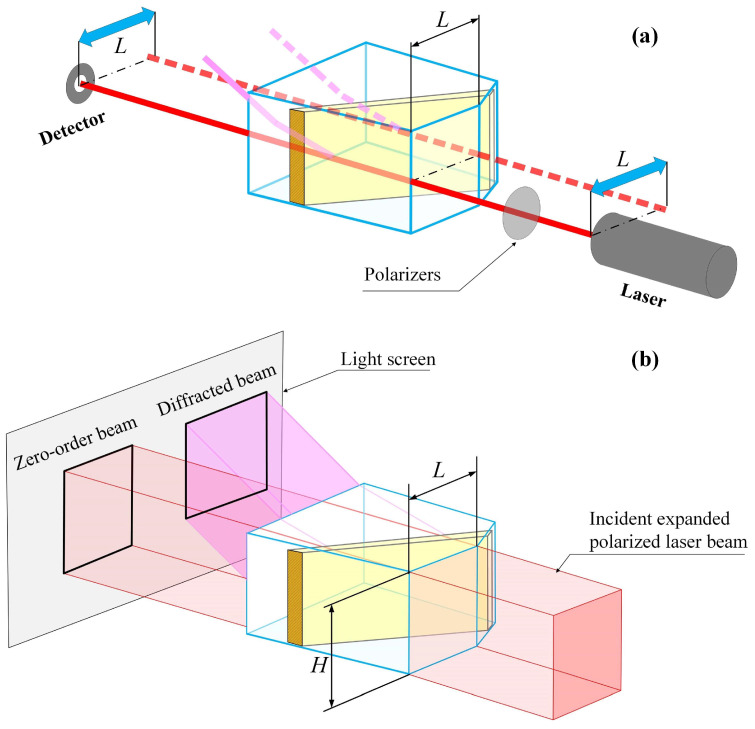
Comparing the single-beam acousto-optic method and the full-aperture acousto-optic method: (**a**) single-beam acousto-optic method; (**b**) full-aperture acousto-optic method.

As illustrated in Figure 3a, conventional acousto-optic testing methodologies assume the acoustic field to be a plane wave. A single laser beam is employed to ascertain the power–distance dependence at the acoustic field’s center, representing the power–distance dependence of the full BAW region.

Nevertheless, the strong acoustic anisotropy of tellurium dioxide crystals results in a redistribution of acoustic power, which is significant for relatively low-frequency ultrasound. This means that the plane wave assumption may be incorrect at low frequencies. The measurement results are due to the simultaneous action of acoustic anisotropy and the attenuation of ultrasound, which makes the acoustic attenuation value unreliable.

The second method proposed in this paper is based on an expanded beam laser, as illustrated in Figure 3b. The acoustic field within the optical aperture is tested using an expanded and collimated laser beam. The full-aperture beam traverses the BAW field, and the non-uniform distribution of the BAW intensity gives rise to diffraction light distributions of varying intensities.

By registering the diffracted light intensity distributions by camera, we can determine the distribution of ultrasound power distributions in the region of AO cells for a given acoustic frequency. This measurement method eliminates the step of multi-distance testing; thus, it is much less laborious and more accurate than the first one. Moreover, two-dimensional acoustic power distribution information can be directly detected.

The most important advantage is the average acoustic power distribution through the full aperture along the acoustic propagation direction. We can almost remove the effect of the acoustic field power redistribution and obtain more reliable information about the attenuation value.

### 2.2. Ultrasound Field Structure Simulation

The acoustic field simulation method applied is based on the improved plane wave angular spectrum approach. The improved angle spectrum method accounts for the influence of medium acoustic anisotropy by assigning different walk-off angles to waves propagating in various directions. The method of acoustic field structure simulation is almost identical to those described in [23,26]. In the calculations, we use the following equation:

(4) $$\begin{array}{l} a(x,z)={\left(\frac{\Omega }{2\pi }\right)}^{2}\int_{-\infty }^{+\infty }A_{0}(\phi S)\times \left[S^{2}+S\left(\phi \frac{\partial S}{\partial \phi }\right)\right]\\ \times \exp\left\{-j\Omega S\left[\phi z+\left(1-\frac{\phi^{2}}{2}\right)x\right]\right\}d\phi , \end{array}$$

Here, ϕ is the small angle being measured from the acoustic beam spatial spectrum axial component in the (1$\bar{1}$0) plane, $A_{0}(\phi S)$ is the acoustic disturbance angular spectrum at the medium input, Ω=2πf is the ultrasound angular frequency, and S(ϕ) is the acoustic slowness. The acoustic beam propagates along the x coordinate (the x axis is collinear with the acoustic vector direction **K**), while the z axis of coordinates transverse to x represents the transducer’s height direction.

The $\left|a(x,z)\right|$ term represents the acoustic field amplitude distribution, and arga(x,z) is the acoustic field phase distribution. We simulate the amplitude structure of the BAW from the input optical facet of the AO cell for a 6.5° cut angle. The simulation is completed under various frequencies and different transducer height configurations.

### 2.3. Simulation Results

The acoustic field structure is contingent upon three factors: the transducer size, the propagation distance, and the acoustic wavelength. The AOTF device tested in this study has a large optical aperture with a transducer height of 20 mm. The frequency range of the transducer is 40–140 MHz, and the acoustic propagation distance inside the crystal is approximately 25 mm. However, most acousto-optic devices have smaller transducer heights [2,21,22]. To facilitate comparisons of the impact of the transducer height on the acoustic field structure, simulations were conducted for AO devices with smaller transducer heights.

Simulations of the acoustic field structure were conducted based on Equation (4), with the following conditions: frequencies of 40, 90, and 140 MHz and transducer heights of 5, 10, and 20 mm. The transducer was positioned on the left side, the simulated sound field propagation distance was 25 mm, and the height of the simulated area was set to four times the transducer height to avoid distortion in the Fourier transform [29]. The sound wave components exceeding the transducer height were minimal, and to better observe the sound field structure, the displayed actual areas were 5 × 25 mm^2^, 10 × 25 mm^2^, and 20 × 25 mm^2^.

**Figure 4:**
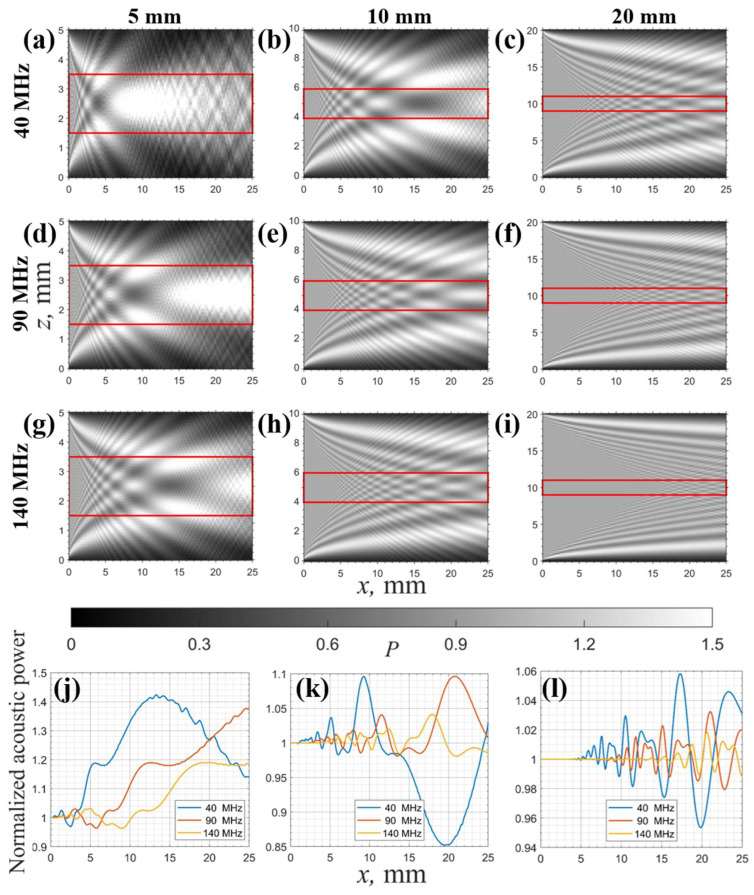
Simulations of the ultrasound field structure field at different frequencies and various transducer heights: (**a**–**i**) 5, 10, and 20 mm transducer heights and the corresponding acoustic power distributions at 40, 90, and 140 MHz, where each column represents a specific transducer height, and each row represents a specific ultrasonic frequency; (**j**–**l**) The relationship between the acoustic power variation and distance for a 2 mm width at the center of the acoustic field.

The simulated device had an ultrasound cut angle of 6.5°, corresponding to an acoustic velocity of 658 m/s. The simulation results are presented in Figure 4a–i, where the acoustic power was normalized to the average power level in the transducer plane and is shown by different brightnesses. To simulate the power redistribution effect of single-laser AO testing, the acoustic power–distance dependence was calculated within a 2 mm wide central region of the simulated acoustic field, as illustrated in Figure 4j,k. The 2 mm central region is marked with a red solid line in Figure 4a–i. Acoustic wave attenuation was not considered in all simulations due to unknown values.

Figure 4a–i reveal that the redistribution of ultrasonic field power arises from the finite size of the transducer (resulting in beam divergence) and is enhanced by the crystal’s acoustic anisotropy. The distribution of acoustic power strongly depends on the ultrasonic frequency and the transducer size; smaller transducers and lower ultrasonic frequencies (corresponding to longer acoustic wavelengths) exacerbate beam diffraction effects, leading to greater non-uniformity in acoustic power.

Figure 4j,k illustrate the impact of a non-uniform acoustic field distribution on the single-beam testing method in the simulation. The presented calculation results (without taking into account acoustic absorption), indicate that for commonly used transducer heights, the single-beam measurement method with low-frequency acoustic fields is significantly affected by the redistribution of ultrasound power. Clearly, this introduces significant errors in acoustic attenuation tests and may lead to a non-monotonic or complex power–distance relationship. Larger transducers also cannot fully mitigate this effect.

In general, for devices with the same crystal cutoff angle, larger transducers and higher-frequency acoustic waves suppress the power non-uniformity caused by acoustic beam diffraction. To mitigate the effects of acoustic field non-uniformity on attenuation test results and to obtain more reliable attenuation data at lower ultrasound frequencies, the use of full-aperture beams to cover the acoustic field is a highly effective approach. This minimizes the effects of acoustic field non-uniformity through averaging over the full acoustic field.

## 3. Experiment and Results

**Figure 5:**
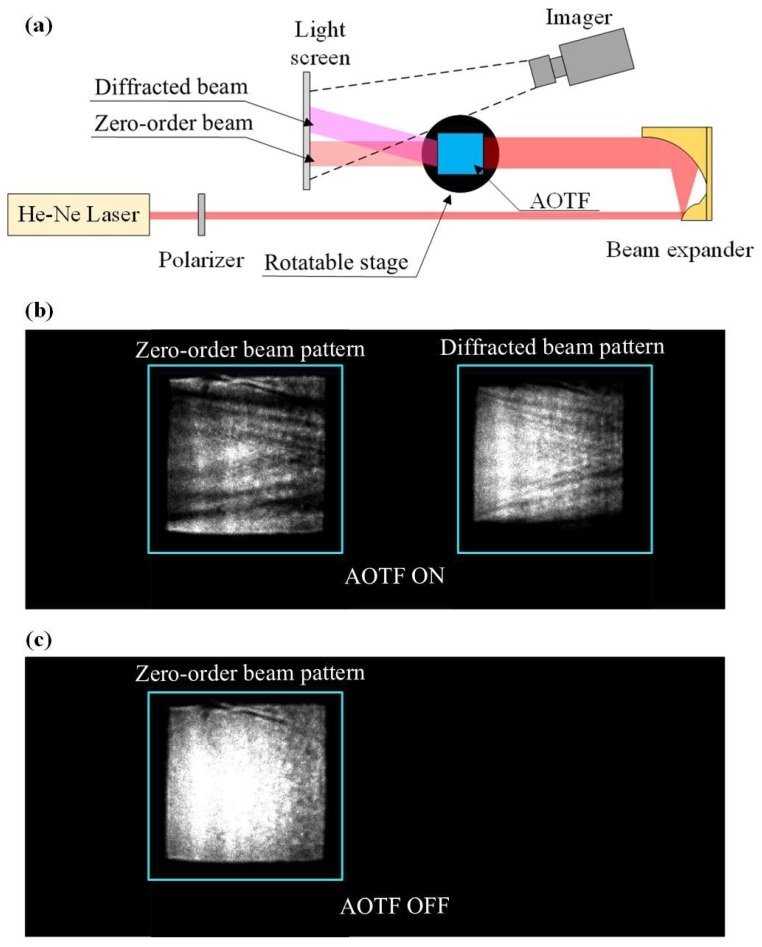
(**a**) Experimental setup. (**b**) Image of zero-order and diffracted patterns (under 50 MHz ultrasound frequency). (**c**) Image of zero-order pattern (AOTF off).

The experimental setup shown in Figure 5a consisted of a He-Ne laser with a polarizer and a reflective beam expander, an AOTF mounted on a rotating platform, and a light screen and imager for real-time capture of the zero-order and diffracted light images.

The diameter of the expanded beam was approximately 30 mm, which was sufficient to fully encompass the input aperture of the tested AOTF (22 × 22 mm^2^). For each ultrasound frequency, the AOTF was rotated to adjust the incident Bragg angle and the polarization direction of the polarizer, as calculated from the results in Figure 2. The light screen and imager position were adjusted for each ultrasound frequency to make sure that the zero-order and diffracted light images were separated in the light screen. The power of the radio-frequency signal that fed the AOTF transducer was chosen in such a way to produce 10–30% diffraction efficiency.

The original images captured by the imager when the AOTF was switched on and off are illustrated in Figure 5b,c. In Figure 5b, the left-hand side shows the zero-order pattern, while the right-hand side shows the diffraction pattern. Software was used to control the imager and AOTF to capture multiple images simultaneously when the AOTF was switched on and off. The diffraction efficiency distribution across the entire aperture was calculated from the intensities of diffracted and zero-order light and subsequently converted to the acoustic power distribution in the AO cells. Equations (2) and (3) were used in this calculation, allowing the acoustic attenuation coefficients to be measured.

### 3.1. Ultrasound Field Structure Validation

**Figure 6:**
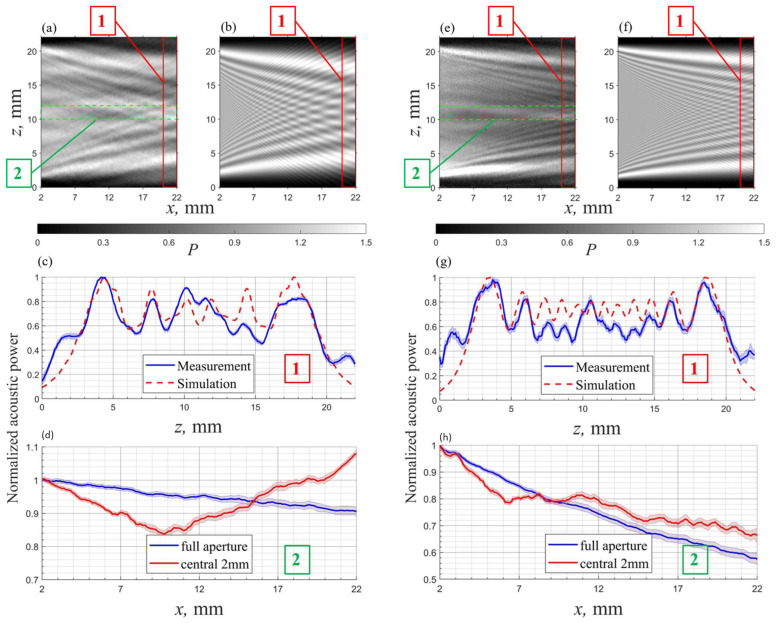
Ultrasound field structure simulation and measurement comparison at two different frequencies: (**a**–**d**) simulation and measurement for a 40 MHz ultrasound field; (**e**–**h**) simulation and measurement for a 90 MHz ultrasound field. (The shaded color regions represent the root-mean-square error ranges of the data.)

The results of the measured and simulated acoustic power distributions in a 22 × 20 mm^2^ (height × length) area at 40 MHz and 90 MHz frequencies are shown in Figure 6a,b,e,f. The acoustic field power was normalized to the average power level in the transducer plane and is shown by differing brightnesses. The simulated and measured acoustic intensity distributions are essentially consistent. The acoustic fields at both frequencies exhibit near-field diffraction patterns, with the beam’s energy narrowing as it propagates and displaying alternating strong and weak regions. The right side of the 90 MHz measured pattern becomes darker due to the rapid attenuation.

To further validate the simulation results, we compared the acoustic power distributions within region 1, which is highlighted by a red solid line in Figure 6a,b,e,f. As illustrated in Figure 6c,g, the peak locations of the acoustic power in both scenarios are nearly identical, and the variation trends are also highly analogous. The 40 MHz acoustic beam width is relatively narrower than the 90 MHz beam width. The observed asymmetry in the measured curve can be attributed to inconsistencies in the transducer input. In Figure 6a,e, it is observed that the sound field intensity at the bottom is consistently higher than that at the top in the transducer height direction. This indicates that the initial sound field power input is uneven and asymmetrical. Furthermore, the reflection of acoustic waves from the top and bottom surfaces also participates in the acousto-optic interaction, resulting in a slight increase in acoustic power at the edges of the measured results.

Furthermore, the power–distance relationship was calculated for the 2 mm center region (representing the single-laser test method) and the full aperture. The position of the center region is marked with a green dashed line in Figure 6a,e, and the results are shown in Figure 6d,h. For the acoustic fields at 40 MHz and 90 MHz, the results for the center region no longer exhibit the typical exponential decay pattern. In contrast, the power–distance relationship for the full aperture at both frequencies closely follows the exponential decay trend. Clearly, data from the center region are influenced by the non-uniform distribution of the acoustic field, with the measured impact being significantly greater than that in the simulation results in Figure 4l. The significantly greater impact in the measurements may be due to uneven transducer input amplitudes. We observe that the sound field power distribution near the transducer on the left side of the image is asymmetrical, indicating that the sound field input is not ideally uniform. In practice, verifying the actual distribution in a small area of the acoustic field is very challenging, as it requires the transducer output to be very close to the ideal state.

Overall, the simulated and measured acoustic power distribution patterns at both lower frequencies are generally consistent. The measured non-uniformity of the sound field is more severe than the simulation suggests, which may be attributed to the non-ideal input of the transducer. Most importantly, the experimental results demonstrate that, compared to the single-beam testing method, the full-aperture testing method provides more reliable acoustic attenuation data. Averaging the acoustic power over the entire beam can reduce the impact of power non-uniformity and other potential non-ideal factors.

### 3.2. Attenuation Measurement

The test ultrasound frequency range was between 40 and 140 MHz, with 10 MHz intervals. The acoustic power distribution for each frequency was averaged along the propagation distance to establish the acoustic power–distance relationship.

**Figure 7:**
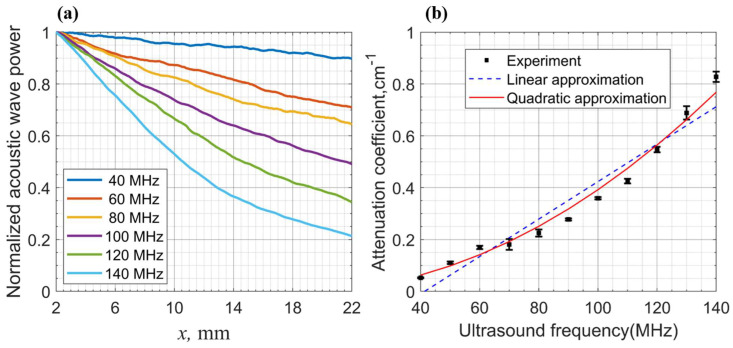
Shear acoustic wave attenuation measurement results: (**a**) Dependency of normalized acoustic wave power on distance from transducer obtained at 40-140 MHz. (**b**) Attenuation coefficient γ dependence on ultrasound frequencies: experimental result dots, with linear and quadratic approximation curves.

Partial results are presented in Figure 7a. The measured acoustic power distances ranging from the transducer were about 2 to 22 mm. The acoustic power values were normalized to the power closest to the transducer for each frequency. These distance values are enough to judge the acoustic field power distribution among most common AO devices. 

At various frequencies, each power–distance dependence exhibits a typical exponential decay shape. Thus, the experimental curve may be approximated by an exponential dependence that looks like

(5) $$P=P_{0}\times \exp(-\gamma x)$$

where $P_{0}$ is the acoustic wave power near the transducer, x is the distance in the crystal measured from the transducer, and *γ* is the acoustic wave attenuation coefficient for the propagation direction in the crystal.

The results of the attenuation coefficient values are presented in Figure 7b. The results indicate that the attenuation coefficient varies significantly as the ultrasonic frequency ranges from 40 to 140 MHz. At 40 MHz, the coefficient is 0.0524 cm^−1^ (0.23 dB/cm), increasing to 0.2774 cm^−1^ (1.20 dB/cm) at 90 MHz and further to 0.8277 cm^−1^ (3.60 dB/cm) at 140 MHz. The attenuation coefficient at 90 MHz is approximately five times that at 40 MHz, and at 140 MHz, it is approximately sixteen times higher than that at 40 MHz.

We attempted to fit the data using both linear and quadratic models (as indicated by the blue dashed and red solid lines in Figure 7b). It is evident that the quadratic model fit more closely. The coefficient employed for the quadratic fit was 3.9203 × 10^−5^ (cm^−1^/MHz^2^) with a standard error of 0.1395 × 10^−5^ (cm^−1^/MHz^2^).

In a comparison with the *γ* values measured previously for AOTFs with an *α* = 7° cutoff angle at higher frequency, our extrapolated empirical data are generally consistent with these findings. In contrast to previous studies, this investigation evaluated acoustic attenuation values in tellurium dioxide crystals at lower frequencies (40–140 MHz) and longer propagation distances, especially highlighting the potential errors in conventional single-beam acousto-optic method testing. These parameters provide accurate data for calculating acoustic power levels in AO devices operating at these frequencies, as well as for addressing issues such as the effective aperture and thermal characteristics due to acoustic losses. Furthermore, the proposed full-aperture acousto-optic testing method is equally applicable to the study of acoustic fields in non-collinear AO devices.

## 4. Conclusions

A full-aperture acousto-optic method is proposed for the direct measurement of the acoustic power distribution along the propagation direction of non-collinear AO devices. This method employs an expanded laser beam that covers the entire aperture of the AO device. By detecting the diffracted light and zero-order light intensity distributions, we can calculate the acoustic power distribution in AO cells.

It was shown that the plane wave assumption of the acoustic field may not be applicable, particularly in scenarios with smaller transducer heights and lower acoustic frequencies, where the acoustic power distribution is significantly non-uniform. In acoustic attenuation testing, traditional single-beam methods are strongly affected by the non-uniformity of the acoustic field. In this case, the full-aperture test method enables the accurate measurement of attenuation by effectively disregarding the non-uniform distribution of the acoustic field intensity.

In addition to its application in acoustic attenuation testing, this method enables the acquisition of power distributions in different regions of the acoustic field, thereby providing a powerful tool for acoustic field-related research. For example, it can identify the most effective or optimal aperture regions for acousto-optic devices, thereby guiding optimal input regions and drive power levels. The measured acoustic attenuation coefficient can be used to accurately estimate the power levels in different regions of acousto-optic devices, enhancing the precision of laser energy control [22,30]. Also, it could support acoustic field-related research, which is necessary to know the exact acoustic field amplitude distribution within various acousto-optic media and even liquid materials [31].

The proposed method determined the ultrasound attenuation for the slow acoustic mode propagating in TeO_2_ crystal along the direction 6.5° to the [110] axis in the (110) plane. The measurements were conducted over a frequency range of 40–140 MHz, during which the power–distance relationships exhibited a classical shape consistent with the expected acoustic attenuation curves. Conversely, when the test results for acoustic power within the central 2 mm region of the acoustic field were employed (simulation of single-laser beam test results), the power–distance relationship curves within the 50–90 MHz range lost their exponential form, and the attenuation coefficient could not be introduced. This confirms the advantage of the proposed method of obtaining more reliable acoustic attenuation coefficients while ignoring the influence of the non-uniform distribution of the acoustic field.

The results of the tests conducted across the 40–140 MHz range indicate that the variation in the value of the attenuation coefficient is closer to a quadratic relationship rather than a linear one with the ultrasonic frequency. The proposed method can also be applied to other AO materials.

## Data Availability

Data are contained within the article.
